# Remarks on the Treatment of Whitlow

**Published:** 1818-04

**Authors:** J. Wood


*For the London Medical and Physical Journal.*

*Remarks on the Treatment of Whitlow;* by J. Wood, M.D.

WITH the view of lessening the sum of human misery, I have one, amongst other memorandums, to make known, a simple plan of treating Whitlow with success, and *without pain.* I was not aware, until I read Dr. Balfour's paper, in the last Number of the London Medical and Physical Journal, that the emollient plan of treating whitlow is now generally laid aside; and, I rather think, this remark will only apply to a few places. It has very frequently happened to me, in my attendance on the diseases in families, that I have been consulted in very bad cases of whitlow, treated with poultices, which I observed did very great mischief; that the formation of pus ceased on laying the poultice aside, and that on dressing the part almost dry, it soon healed. I was led from this, to try to lessen the formation of pus in the first instance, or to render the *quantity formed as small as possible;* and, in this, I succeeded merely by the frequent application of cold vinegar and water, equal parts, in the beginning of the inflammation, and wrapping the finger up afterwards in linen, applying a little sweet oil to it after the vinegar and water. It thus frequently happened, that the whitlow disappeared, or that there was a small collection of matter under the skin, which was let out by the point of a lancet, as Dr. Balfour mentions, and the finger wrapped up with a piece of dry linen immediately healed. Two days, or seldom more than three, completed the process and the cure. Whitlow has often occurred amongst the servants in my own house, and the servants of other houses where I attend, and this plan never fails to give relief, and remove the effects in a day or two.

Dr. Balfour says, "*Si quid novesti* rectius *istis, candidus imperti;*" I will not assert, that the plan I have mentioned is *better* than Dr. Balfour's: I will say, that it is less painful to the patient, less troublesome to the operator, and more likely to be assented to than Dr. Balfour's. In one of Dr. B's cases, vinegar was applied by the patient herself; (it is Case 1st.)

I cannot but think, that, in many parts of the country, poultices are yet applied to whitlow, with all their consequent misery, and tedious bad effects; and, I trust, that they will now be totally given up, as there is scarcely a medical practitioner in the kingdom that has not the advantage of seeing the contents of the London Medical and Physical Journal.

*Newcastle-upon-Tyne;*

*March* 5, 1818.

N.B.—Vinegar is often found so weak as not to require dilution with water. After vent is given by the point of a lancet to any matter that has formed, the skin that has been separated by the matter must be allowed to remain till another skin is formed, when the former becomes dry, and falls off.

---

*For the London Medical and Physical Journal.*

